# Reinfection and ceftriaxone tolerance in a clinical case of recurrent gonorrhoea: a case report supported by *in vitro* and *in vivo* models

**DOI:** 10.3389/fcimb.2026.1720396

**Published:** 2026-04-23

**Authors:** Izumo Kanesaka, Saïd Abdellati, Thibaut Vanbaelen, Irith De Baetselier, Tessa de Block, Basil Britto Xavier, Chris Kenyon, Sheeba Santhini Manoharan-Basil

**Affiliations:** 1 Department of Clinical Sciences, Institute of Tropical Medicine Antwerp, Antwerp, Belgium; 2 Department of Infection Control and Prevention, Faculty of Nursing, Toho University, Tokyo, Japan; 3 Department of Medical Microbiology and Infection Prevention, University Medical Center, Groningen, University of Groningen, Groningen, Netherlands; 4 University of Cape Town, Cape Town, South Africa

**Keywords:** ceftriaxone, MDK99, *Neisseria gonorrhoeae*, reinfection, tolerance, treatment failure

## Abstract

**Background:**

Antibiotic tolerance slows bacterial killing during drug exposure without changing minimum inhibitory concentration (MIC) and is often missed by MIC-based testing in *Neisseria gonorrhoeae*.

**Objectives:**

To quantify ceftriaxone tolerance using complementary laboratory assays and to integrate these readouts with genomic typing to interpret recurrent urethritis within one patient series.

**Methods:**

Four clinical isolates obtained between 2022 and 2025 underwent tolerance disc testing. From the most recent isolate (25355), tolerant and non-tolerant variants were derived for functional study, including growth curves (log_10_ CFU/mL), minimum duration needed to kill 99% of cells (MDK99) across ceftriaxone concentrations, and a *Galleria mellonella* infection model. Whole-genome sequencing with in silico typing compared strain type between episodes.

**Results:**

Tolerance disc testing was positive for isolates 22073 and 25355. Across the growth trajectory, tolerant variants showed consistently lower bacterial counts than non-tolerant variants. Tolerant variants tended to exhibit longer MDK99 values, and in *Galleria* they declined more slowly under ceftriaxone exposure. Sequencing revealed distinct sequence types across episodes, supporting reinfection rather than within-host persistence.

**Conclusions:**

Assays that capture killing kinetics detected ceftriaxone tolerance that was not captured by MIC-based testing. Genomic analysis distinguished reinfection from persistence. This integrated workflow may improve the evaluation of suspected treatment failure in gonorrhoea.

## Introduction

Antibiotic tolerance is defined as a reduction in the rate of killing during antibiotic exposure without a change in the minimum inhibitory concentration (MIC) (Brauner et al., 2016). In practice, tolerant populations require longer exposure to achieve eradication, and this property can be quantified with time-to-kill metrics such as the minimum duration needed to kill 99% of cells (MDK99) (Brauner et al., 2016). Tolerance is related to, but distinct from, persistence. In persistence, most cells are killed at a rate consistent with susceptibility while a small subpopulation, typically less than one percent, survives. Both phenomena can reflect population heterogeneity without a shift in MIC, but persistence refers specifically to the survival of a small subpopulation during antibiotic exposure (Brauner et al., 2016; Lewis, 2010).

Evidence for tolerance has been described in more than twenty bacterial species and has been associated with difficult to treat infections caused by pathogens such as *Staphylococcus aureus*, *Escherichia coli*, and *Mycobacterium tuberculosis* (Brauner et al., 2016; Levin-Reisman et al., 2017; Santi et al., 2021). Beyond immediate therapeutic challenges, tolerant subpopulations can survive antibiotic exposure and form a reservoir in which resistance mutations may arise and be selected, thereby linking tolerance to the longer-term evolution of antimicrobial resistance (Santi et al., 2021; Levin-Reisman et al., 2017). These observations have prompted interest in routine methods that capture killing kinetics in addition to conventional MIC based testing (Brauner et al., 2016).

In *Neisseria gonorrhoeae*, experimental studies demonstrate that ceftriaxone tolerance can be induced by repeated cycles of drug exposure followed by regrowth, and tolerant phenotypes have been detected among clinical isolates (Balduck et al., 2022). Surveys suggest that the apparent prevalence of tolerance may vary by anatomical site, which could reflect differences in local ecology and drug exposure (Balduck et al., 2024). Recent transcriptomic work has implicated a ribosomal program with downregulation of ribosome-related genes in tolerant phenotypes, consistent with a slowed growth state that is less susceptible to rapid killing (Manoharan-Basil et al., 2025). Together, these data indicate that tolerance in gonococci is measurable *in vitro* and biologically meaningful.

The clinical significance of gonococcal tolerance, however, remains incompletely defined. MIC testing is central to clinical decision making, yet it does not capture killing rate (Unemo and Shafer, 2014). Only one detailed case report to date has linked presumed ceftriaxone tolerance with repeated treatment failure despite susceptible MICs, where prolonged ceftriaxone ultimately achieved cure (Kanesaka et al., 2025). Distinguishing persistence of a tolerant strain from repeat reinfection is a further challenge in clinical practice and requires integration of epidemiologic assessment with genomic typing alongside laboratory assays that characterize tolerance.

We report a patient with recurrent gonococcal urethritis in whom ceftriaxone tolerance was detected. We outline the complementary laboratory approaches used to characterize tolerance, including growth curves, MDK99 measurements, and a *Galleria mellonella* infection model, and we describe genomic analyses undertaken to help distinguish persistence from reinfection. Our aim is to provide a pragmatic framework for evaluating suspected treatment failure in gonorrhoea, to clarify when tolerance may be clinically relevant even when MICs indicate susceptibility, and to motivate future work on how killing-kinetic assays could inform research, surveillance, and eventually clinical care.

## Materials and methods

### Case report

In early 2025, a cisgender man in his twenties presented with four episodes of symptomatic urethritis over a three-month period. Urethral samples were collected at each presentation for diagnostic testing. Clinical information on sexual partners, prior gonococcal infection and vaccination history was obtained via patient consultation and review of medical records.

### Clinical microbiology testing

Urethral swabs were tested using a validated nucleic acid amplification test (NAAT; Roche Cobas 5800 system; Roche Diagnostics, Branchburg, New Jersey), following the manufacturer’s instructions. For culture, specimens were inoculated onto GC selective agar supplemented with 1% IsoVitaleX (BD, Franklin Lakes, NJ, USA) and incubated at 36 °C in 6% CO_2_. Minimum inhibitory concentrations (MICs) for ceftriaxone, azithromycin, and ciprofloxacin were determined. Briefly, testing was performed on GC agar base (BD) supplemented with 1% IsoVitaleX. Colonies from overnight growth were suspended in phosphate buffer saline (PBS) to a 0.5 McFarland standard, spread onto GC agar to obtain a confluent lawn and the gradient E-test (BioMérieux) were placed. After 20-24h incubation, at 36 °C in 6% CO_2_ MICs were measured at the point where the inhibition ellipse intersected the scale. Clinical categorization followed EUCAST breakpoints (EUCAST v13.0, 2023). Quality control was performed according to EUCAST recommendations. Cultures were stored at -80 in skim milk with 30% glycerol.

### Strains used in this study

The patient had a total of 12 documented episodes of gonococcal infection from 2018-2025, of which culture was available from four occasions (**Table 1**). Out of the four urethritis episodes in early 2025, *Neisseria gonorrhoeae* isolates were recovered successfully from two urethral swabs (isolate ids: 25355 and 25322). To provide a longitudinal context, we also included cultures from earlier infections. Therefore, in total four isolates were included in this study: 22073 (February 2022), 24038 (January 2024), 25322 (April 2025) and 25355 (May, 2025). Additional functional *in vitro* and *in vivo* assays, including growth curves, determination of the minimum duration for killing 99% of the population (MDK99), and infection experiments using the *Galleria mellonella* model were conducted on tolerant and non-tolerant subpopulations derived from isolate 25355. This isolate was chosen for detailed functional analysis because it was the most recently recovered isolate and yielded both tolerant and non-tolerant subpopulations, allowing direct phenotypic comparison within the same clinical isolate background.

**Table 1:** Isolates used in the study.

Date of isolation	PCR NG	Culture	Isolate ID	MIC (µg/mL)			WGS	ST-Type	TD test
				Azithromycin	Ceftriaxone	Ciprofloxacin			
2018	Positive	NA	NA	ND	ND	ND	ND	ND	ND
2020/7/10	Positive	NA	NA	ND	ND	ND	ND	ND	ND
2020/11/9	Positive	NA	NA	ND	ND	ND	ND	ND	ND
2021/4/14	Positive	NA	NA	ND	ND	ND	ND	ND	ND
2021/8/3	Positive	NA	NA	ND	ND	ND	ND	ND	ND
2022/2/17	Positive	Yes	22073_non-tolerant 22073_tolerant	3	0.016	0.023	Yes Yes	11422	Positive
2023/10/16	Positive	NA	NA	ND	ND	ND	ND	ND	ND
2024/1/15	Positive	Yes	24038_non-tolerant	0.5	0.006	1	Yes	7827	Negative
2025/4/23	Positive	Yes	25322_non-tolerant	0.094	0.003	0.002	Yes	18166	Negative
2025/5/8	Positive	Yes	25355_non-tolerant 25355_tolerant	1.5	0.012	4	Yes	10314	Positive
2025/5/15	Positive	NA	NA	ND	ND	ND	ND	ND	ND
2025/6/5	Positive	NA	NA	ND	ND	ND	ND	ND	ND

NA, not available; ND, not determined.

### Tolerance was detected in isolates 22073 and 25355

Tolerance Detection (TD) assays were performed as previously described (Balduck et al., 2022) on all four isolates (22073, 24038, 25322 and 25355). Briefly, colonies from each isolate were suspended in GC broth to a turbidity equivalent to a 0.5 McFarland standard and inoculated onto GC agar plates supplemented with 1% IsoVitaleX. A ceftriaxone (CRO) disc (0.016 µg) was placed on the agar surface for 24 h at 36 °C in 6% CO_2_. After this exposure, the CRO disc was replaced with a nutrient disc, and plates were further incubated for 48 h. Regrowth of colonies within the inhibition zone was recorded as a positive TD result.

### Growth curve assay

Strains (25355 tolerant and non-tolerant) were suspended in GC broth supplemented with IsoVitaleX at a final concentration of 10³ CFU/mL and incubated at 36 °C in a 6% CO_2_ atmosphere under static conditions. Colony counts were determined at 0, 2, 4, 6, and 8 h by plating 100 fold serial dilutions on GC agar (Becton Dickinson). After incubation at 36 °C for 24–48 h, colonies were counted and the mean log_10_ CFU/mL from three replicates was calculated. Results were summarized as means of three independent experiments. Pairwise comparisons at each time point were performed using Student’s t tests (two sided). In addition, to assess overall differences across the growth trajectory, we analyzed log_10_ CFU/mL using a two way model with factors “strain type” (tolerant vs non-tolerant) and “time” (0, 2, 4, 6, 8 h), with cluster robust standard errors (clustered by biological replicate) to account for repeated measures.

### Quantification of ceftriaxone tolerance using MDK99 assay

To quantify ceftriaxone tolerance, the minimum duration required to kill 99% of the bacterial population (MDK99) was determined using a modified version of a previously established protocol (Brauner et al., 2016). Two populations of *N. gonorrhoeae* were analyzed: (i) ceftriaxone-tolerant colonies originating from isolate 25355, and (ii) non-tolerant colonies from the same isolate. Bacterial suspensions were adjusted to yield approximately 200 CFU per well in 96-well plates containing 200 µL of GC broth supplemented with 1% IsoVitaleX. The bacteria were treated with ceftriaxone at final concentrations of 0.004, 0.008, 0.016, and 0.032 µg/mL for various time intervals (2, 3, 4, 5, 6, 8, and 24 hours). After drug exposure, wells were washed three times with 200 µL of PBS to remove residual antibiotic, and then refilled with fresh GC broth containing 1% IsoVitaleX. The plates were incubated overnight at 36 °C under 6% CO_2_. On the following day, turbidity was assessed visually. The MDK99 was defined as the minimum exposure time after which no visible regrowth was observed in any replicate. Each condition was tested in triplicate across three independent experiments. Data were reported as mean ± standard deviation (SD). For statistical analysis, Welch’s t-test (unpaired, assuming unequal variances) was used to compare MDK99 values between strains at each drug concentration. All statistical analyses were performed using GraphPad Prism version 10 (GraphPad Software, San Diego, CA, USA).

### *In vivo* tolerance assay using Galleria mellonella

An *in vivo* tolerance assessment was performed using the *Galleria mellonella* larval infection model to investigate the ability of ceftriaxone-tolerant *N. gonorrhoeae* strains to survive under antibiotic exposure (Tsai et al., 2016). Larvae were challenged with the tolerant variant of strain 25355 and the non-tolerant variant of strain 25355. Bacterial cultures were prepared to match an 8 McFarland turbidity standard, and 30 µL of the suspension (approximately 2.4 × 10^9^ CFU/mL) was injected into the hemocoel via the left rear proleg using a Hamilton syringe. Ten minutes post-infection, ceftriaxone was administered by injecting 10 µL of a 0.016 µg/mL solution into the opposite (right) proleg. Infected larvae were then incubated at 36 °C. At 2, 4, 6, and 8 hours after infection, approximately 50 µL of hemolymph was extracted from each larva, serially diluted, and plated onto GC selective agar. Plates were incubated at 36 °C for 24–48 hours to allow colony growth. Colonies recovered were confirmed as *N. gonorrhoeae* via matrix-assisted laser desorption/ionization time-of-flight mass spectrometry (MALDI-TOF MS). Each group was tested in triplicate. Bacterial load differences between strains at each time point were evaluated using Welch’s t-test (two-sided, unequal variances). All statistical analyses were carried out using GraphPad Prism version 10 (GraphPad Software, San Diego, CA, USA). Ethical approval was not required for this part of the study as *Galleria mellonella* is an invertebrate and not subject to institutional review board (IRB) or animal ethics committee oversight.

### Whole genome sequencing, assembly and variant analysis

In total six isolates were outsourced to Eurofins Genomics (Germany) for DNA isolation, library preparation and sequencing. These included the 4 non-tolerant isolates (22073, 24038, 25322 and 25355) and two tolerant isolates from 25355 and 22073. DNA libraries were prepared using the Stranded TruSeq DNA library preparation kit (Illumina Inc., San Diego, CA, USA). Sequencing was performed on a NextSeq 6000 platform using v2 chemistry to generate 2 X 150 bp paired-end reads. Raw Illumina reads were subjected to quality control using FastQC (v0.11.9) (Andrews, 2010). Trimming of the raw reads was performed using Trimmomatic (v0.39) (Bolger et al., 2014) with the following parameters: leading and trailing base quality cutoff of 3, sliding window trimming with average quality 15 across 4 bases, and minimum read length of 30 bases. The trimmed raw reads were *de novo* assembled using SPAdes (v3.14.0) with the parameters --trim --depth 150 --careful (Prjibelski et al., 2020). All the draft genomes were annotated using Prokka (v1.14.6) (Seemann, 2014) and were uploaded to Pathogenwatch (v3.0.2) (Argimón et al., 2021) for *in silico* genotyping and antimicrobial resistance (AMR) prediction. Sequence types were determined using MLST (PubMLST) (Jolley and Maiden, 2010), NG-STAR (Demczuk et al., 2017), and NG-MAST schemes (Martin et al., 2004). For variant analysis, trimmed Illumina reads from isolates 25355-tolerant and 22073-tolerant were mapped against the non-tolerant counterparts using BWA-MEM (Jung and Han, 2022) and single nucleotide polymorphisms (SNPs) were determined using freebayes implemented in Snippy (v4.6.0; Seemann, 2015) with default parameters (10× minimum read coverage and 90% read concordance at the variant locus).

### Genome assembly quality and completeness

Genome assembly quality and completeness were assessed using complementary approaches. Assembly statistics, including contiguity and genome size metrics, were evaluated using QUAST (Gurevich et al., 2013). Genome completeness was assessed using BUSCO (v5) with the neisseriales_odb12 dataset (Manni et al., 2021). In addition, genome completeness and contamination were independently evaluated using CheckM2 (Chklovski et al., 2023).

All analyses were performed using default parameters unless otherwise specified.

### Data availability

The raw reads are available under NCBI BioProject: PRJNA1334155.

### Ethics and consent

We obtained written informed consent from the patient to report anonymized clinical details. All identifying information was removed in accordance with institutional policy and the principles of the Declaration of Helsinki. This case report was reviewed by the local ethical standards and was considered exempt from formal ethics committee approval, as all investigations were conducted as part of routine clinical care. Experiments involving *Galleria mellonella* (invertebrate model) do not require institutional animal ethics approval at our institutions.

## Results

In early 2025, a cisgender man in his twenties presented with four episodes of symptomatic urethritis over a 3-month period. In each case he had a visible urethral discharge and *N. gonorrhoeae* was detected on urethral NAAT and confirmed by culture where available. Out of the four episodes*, N. gonorrhoeae* was cultured successfully on two occasions (isolate IDs: 25355 and 25322). The ceftriaxone MICs of all isolates were low (**Table 1**).

**Figure 1:**
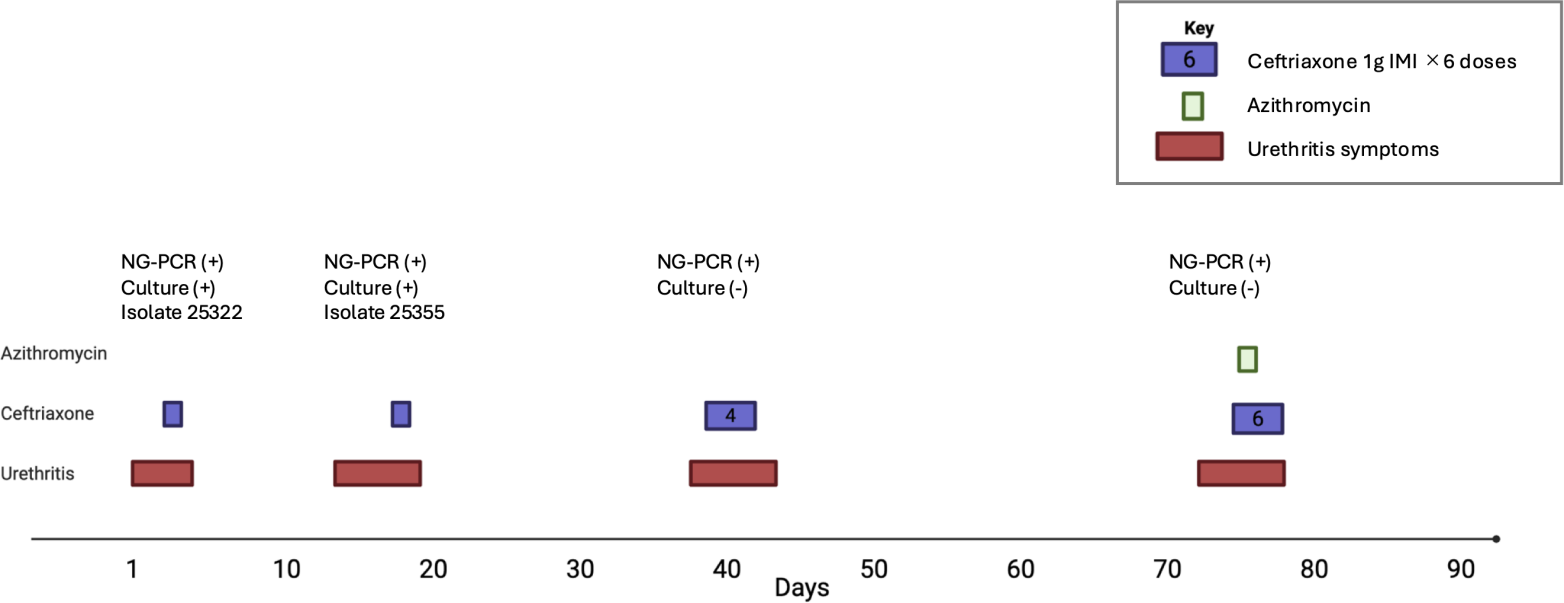
Clinical timeline of a patient with recurrent gonococcal urethritis. Episodes occurred in early 2025 and were managed with ceftriaxone (purple boxes) alone or in combination with azithromycin (green boxes). Urethritis symptoms are shown in red. NG-PCR positivity is indicated for each episode. Cultures were positive in two episodes, yielding isolates 25322 and 25355 for subsequent phenotypic and genomic analyses, whereas the later NG-PCR-positive episodes were culture-negative.

We denote the day of treatment for the first episode as day 0. Treatment regimens across the four episodes are shown in **Figure 1**. On the first two occasions he received ceftriaxone 1 g IM; on the third, four doses were administered over 7 days (days 0, 1, 4, 7); and symptoms improved after each course.

He reported sexual contact with four other men in the three months prior to day 0. He had no regular partners and his last sexual contact was 4 days prior to day 0. He reported that he had had no sexual contact since then. He took HIV preexposure prophylaxis on demand and seldom used condoms. He had no known exposure to objects that could transmit *N. gonorrhoeae* via fomite transmission. Between 3 months and 8 years prior to day 0, he had 12 documented episodes of gonococcal infection, of which culture results were available for four (isolate ID: 22073 in February 2022, isolate ID: 24038 in January 2024, and isolate IDs: 25322 and 25355 in 2025). He had received two doses of 4CMenB vaccine (Bexsero^®^) in late 2024.

His fourth episode on day 76 was treated with 6 doses of 1 g ceftriaxone IMI (days 0, 1, 2, 3, 4, 5, 7) plus azithromycin 1 g PO 12 hourly for 24 hours. He had rapid resolution of his symptoms and these have not returned. A *N. gonorrhoeae* NAAT of a pooled urine/rectal/pharyngeal sample was negative on day 112.

### Genome assembly quality and completeness

Genome assembly quality and completeness were assessed using complementary approaches, including BUSCO (**Supplementary Table 1**), QUAST (**Supplementary Table 2**), and CheckM2 (**Supplementary Table 3**). BUSCO analysis using the neisseriales_odb12 dataset demonstrated consistently high completeness across all assemblies, ranging from 95.6% to 96% complete BUSCOs. Fragmented BUSCOs were low across all genomes (0.6-0.8%), and the proportion of missing BUSCOs remained limited (3.2-3.5%), indicating overall high assembly integrity. These metrics were consistent across both tolerant and non-tolerant variants, with no systematic differences observed between paired isolates. Independent assessment using CheckM2 further supported these findings, confirming high genome completeness with minimal contamination across all assemblies. Assembly statistics generated by QUAST were consistent with expected genome characteristics for *N. gonorrhoeae.*

### Whole-genome sequencing indicated reinfection rather than persistence

Sequence based typing revealed that isolate 22073 (both non-tolerant and tolerant), 24038, 25322 and 25355 (both non-tolerant and tolerant) belonged to ST11422, ST7827, ST18166 and ST10314, respectively. NG-MAST analysis showed partial discriminatory power; the *por* gene were assigned to 3 allele types [1808 (ST 7827), 908 (ST11422) and 6720 (ST 10314 and ST 18166)], while *tbpB* alleles were either 5, 29 or non-typeable (“new”).

PathogenWatch predicted resistance to azithromycin in all isolates except 24038 and 25322; resistance to ciprofloxacin in all isolates except 22073 and 25322; and resistance to sulfonamides across all isolates. These predictions were supported by screening against CARD and MEGARes. A core set of efflux-associated determinants (*mtrCDE*, *macAB*, *farAB*/*farR*) was present in all isolates. No determinants associated with decreased susceptibility to ceftriaxone or cefixime were detected, consistent with the low ceftriaxone MICs observed phenotypically. WGS revealed a high degree of overall genomic similarity between tolerant and non-tolerant variants derived from the same isolate, with no evidence of large-scale chromosomal rearrangements or clear genome-wide divergence. No SNPs were detected between the tolerant and non-tolerant subpopulations derived from the same isolates.

### Tolerance was detected in isolates 22073 and 25355

TD assays were conducted on all four available isolates (22073, 24038, 25322, and 25355). The earliest isolate 22073 (collected in 2022) tested positive in the Tolerance Detection (TD) assay, indicating the presence of a ceftriaxone-tolerant subpopulation. In contrast, the isolates 24038 (2024) and 25322 (2025) were TD-negative. The more recent isolate from 2025 (isolate id: 25355) was TD positive. From this isolate, both tolerant and non-tolerant subpopulations were derived and subsequently subjected to *in vitro* and *in vivo* analyses.

### Tolerant isolate (25355) maintained lower bacterial counts throughout the growth assay

**Figure 2:**
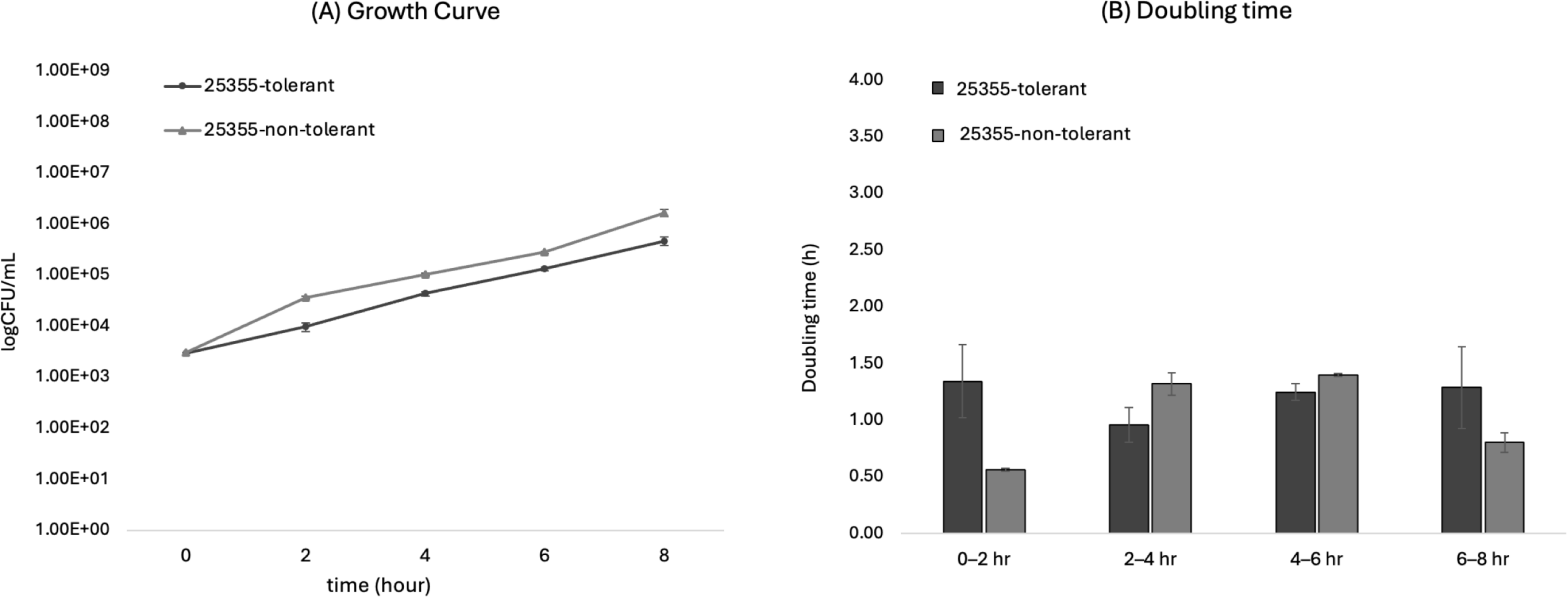
Growth curves and doubling time analysis of *N. gonorrhoeae* strains. **(A)** Growth curves of 25355 (tolerant and non-tolerant) strains in GC broth. Bacterial growth was measured at the indicated time points and is shown as log10 CFU/mL (mean ± SD of three independent experiments). A two-way model (strain type × time; cluster robust SEs) detected a significant overall group effect (F = 66.56, p = 1.6 × 10^-4^) and a significant interaction (F = 95.69, p = 6.5 × 10^-5^), whereas pairwise t-tests at individual time points were not significant (all p > 0.05). **(B)** Doubling times of each strain calculated for four different intervals based on the growth curve data shown in **(A)**. Error bars indicate SD across three independent biological experiments.

Both the tolerant and non-tolerant variants of isolate 25355 entered logarithmic growth under standard culture conditions. Differences in bacterial counts were observed from 2 hours onward, with the tolerant variant consistently showing lower CFU per mL than the non-tolerant variant. Pairwise t tests at individual time points did not reach statistical significance (all p > 0.05). However, when analyzing the entire trajectory with a two way model (strain type × time; cluster robust standard errors), we detected a significant overall group effect (F = 66.56, p = 1.6 × 10^-4^) and a significant interaction (F = 95.69, p = 6.5 × 10^-5^), indicating that tolerant variants maintained lower counts across time and that the magnitude of this difference varied with time. Baseline values at 0 h did not differ (p = 0.367) (**Figure 2A**). Doubling time analysis based on these curves did not show significant differences between the variants (**Figure 2B**).

### Tolerant isolate (25355) tended to show longer MDK99 under ceftriaxone exposure

**Figure 3:**
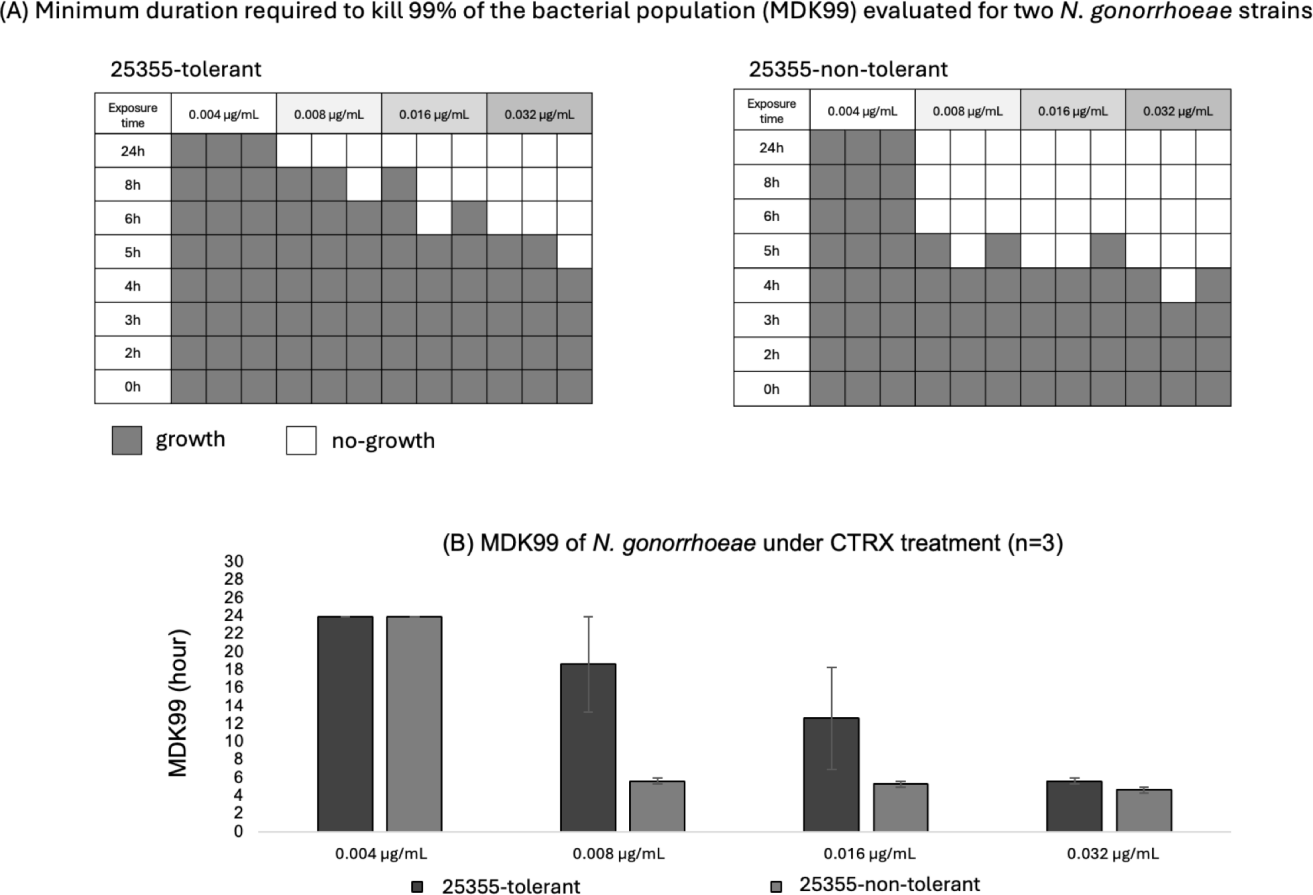
MDK99 of *Neisseria gonorrhoeae* under ceftriaxone treatment. **(A)** Minimum duration required to kill 99% of the bacterial population (MDK99) evaluated for two *N. gonorrhoeae* strains (25355-tolerant, 25355-non-tolerant) exposed to ceftriaxone at concentrations ranging from 0.004 to 0.032 µg/mL. Gray squares indicate conditions where regrowth was observed; white squares indicate no detectable growth. **(B)** Mean MDK99 values from three independent biological experiments are shown as bar graphs, with error bars indicating SD. Black bars indicate 25355-tolerant and grey bars indicate 25355-non-tolerant.

The minimum duration required to kill 99% of the bacterial population (MDK99) was assessed for the tolerant and non-tolerant variants of isolate 25355 across ceftriaxone concentrations ranging from 0.004 to 0.032 µg/mL. The tolerant variant consistently exhibited prolonged survival compared to the non-tolerant variant. Although there was an apparent difference in MDK99 values between the two populations, these differences did not reach statistical significance (**Figure 3**).

### In the *Galleria mellonella* model, the tolerant isolate (25355) exhibited longer survival under ceftriaxone treatment

**Figure 4:**
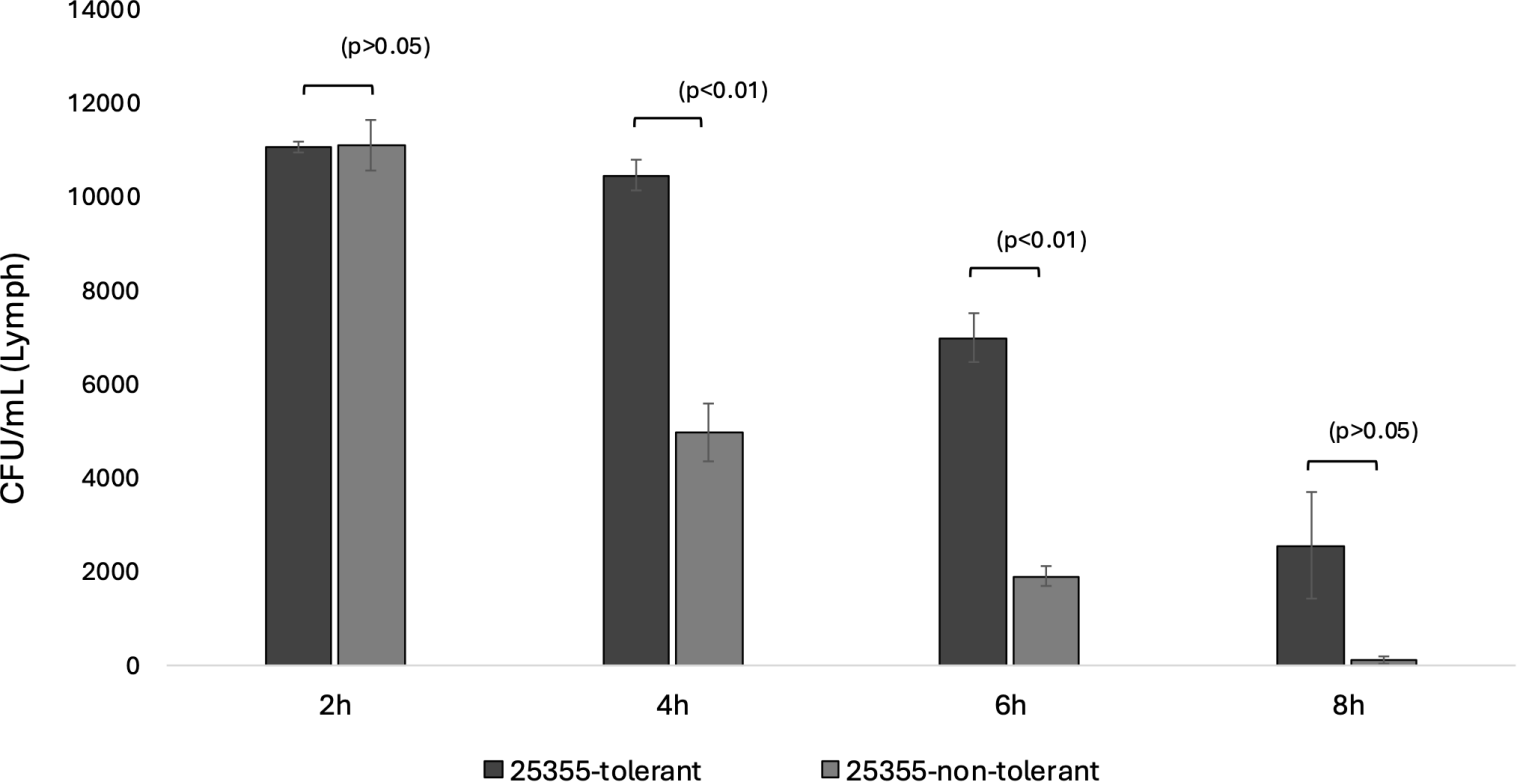
Survival of *N. gonorrhoeae* strains in larvae following ceftriaxone (CTRX) treatment (0.016 µg/mL). *Galleria mellonella* larvae were infected with *N. gonorrhoeae* strain 25355 (tolerant and non-tolerant variants), and ceftriaxone (0.016 µg/mL) was injected 10 minutes post-infection. Bacterial loads in the hemolymph were quantified at 2, 4, 6 and 8 hours by plating on GC selective agar. Bars show mean CFU/mL from individual larvae (n=3); error bars indicate standard deviation. Dotted brackets denote pairwise comparisons between tolerant and non-tolerant variants at each time point using Welch’s two-sided t-test with unequal variances. Statistical significance is indicated above the brackets (p < 0.01); values with p > 0.05 were considered not statistically significant.

To assess *in vivo* survival under ceftriaxone exposure, *Galleria mellonella* larvae were infected with the tolerant and non-tolerant variants of isolate 25355 and treated with ceftriaxone (0.016 µg/mL). Bacterial counts in hemolymph were determined at 2, 4, 6 and 8 hours post-infection. The non-tolerant variant showed a reduction in bacterial counts at 2 hours, with further decline observed at later time points. In contrast, the tolerant variant did not show a marked reduction up to 4 hours and maintained higher bacterial loads compared to the non-tolerant variant. Statistical analysis confirmed significant differences between tolerant and non-tolerant variants at 4 and 6 hours (Welch’s t-test, *p* < 0.01), whereas differences at 2 and 8 hours were not statistically significant (*p*>0.05) (**Figure 4**).

## Discussion

Antibiotic tolerance, defined as a reduced rate of killing without a change in MIC, has been implicated in poor outcomes for several pathogens, but its clinical role in *Neisseria gonorrhoeae* remains incompletely defined (Brauner et al., 2016). In our laboratory study, we showed that ceftriaxone tolerance can be experimentally induced by repeated exposure, operationalised as serial cycles of drug challenge followed by regrowth without antibiotic, and quantified with metrics of killing kinetics such as MDK99 (Balduck et al., 2022). We also detected tolerance among clinical isolates in that work. Complementing these findings, our first clinical case report (Kanesaka et al., 2025) linked ceftriaxone tolerance to delayed clearance that responded to prolonged therapy. Together, these studies indicated that tolerance may be clinically relevant even when isolates test susceptible by methods based on MIC.

Against this background, the present case initially appeared to represent persistence of a ceftriaxone tolerant strain during recurrent urethritis. Tolerance was indeed detected during the course, including in the final culture positive isolate, and phenotypic assays demonstrated hallmark features of tolerance. When we compared the tolerant and non-tolerant variants derived from isolate 25355, the tolerant variant showed slower bactericidal kinetics *in vitro* and prolonged survival *in vivo* in the *Galleria mellonella* model. In that model, burdens of the non-tolerant variant declined significantly from early time points, whereas the tolerant variant did not show a significant reduction through 4 hours and maintained higher counts later. These results align with the concept that tolerance can impair killing dynamics under drug exposure without altering MIC (Brauner et al., 2016). However, no clear genomic differences were identified between the tolerant and non-tolerant variants in our whole-genome comparison. This suggests that the observed phenotype may not necessarily be explained by fixed chromosomal mutations alone. Alternative explanations should therefore be considered, including stochastic phenotypic variation, phase variation, differences in inoculum density, and effects related to isolation or culture conditions. In line with this, emerging evidence indicates that ceftriaxone tolerance in *N. gonorrhoeae* can arise despite high genomic similarity and may instead be associated with transcriptional reprogramming and localized genetic variation, including at pilin-associated loci (Kanesaka et al., 2026). Together, these observations support a model in which tolerance reflects dynamic regulatory and phenotypic states rather than stable genome-wide sequence divergence, although subtle genetic or regulatory contributions cannot be fully excluded.

Crucially, whole genome sequencing of consecutive isolates showed different sequence types, demonstrating that each episode was caused by genetically distinct strains. Thus, while tolerance was present and biologically meaningful, the most parsimonious explanation for recurrence in this patient is repeat reinfection rather than persistence of a single tolerant clone. This underscores the critical role of WGS in distinguishing treatment failure from reinfection, an assessment that cannot be made using phenotypic data alone. In this sense, the current case refines the clinical picture established in our first case report (Kanesaka et al., 2025): tolerance can contribute to delayed killing and may influence outcomes, yet recurrent disease may still be driven primarily by reinfection. The present case therefore suggests that tolerance and reinfection are not mutually exclusive concepts, with tolerance influencing bacterial killing in individual isolates while the overall clinical pattern is explained more convincingly by repeated acquisition of distinct strains.

These observations yield two practical implications. First, reinfection is common in individuals with repeated gonorrhoea even when therapy is appropriate and MICs indicate susceptibility. Clinical management should therefore combine antimicrobial treatment with prevention measures, partner notification, and timely retesting (Unemo and Shafer, 2014; Unemo et al., 2019; Barbee et al., 2022). Second, tolerance remains clinically relevant but is invisible to conventional MIC-based testing. MDK type assays that capture killing kinetics, as used in our laboratory work, offer a way to quantify tolerance in research settings and surveillance and may help identify cases in which tolerance could compromise cure, for example under suboptimal drug exposure or high bacterial inocula (Brauner et al., 2016; Levin-Reisman et al., 2017; Santi et al., 2021). However, TD-based assays may also be influenced by experimental conditions, and factors such as inoculum density, antibiotic carryover, or phase-variable phenotypic states may occasionally affect the interpretation of positive tolerance results.

This case also illustrates a pragmatic evaluation pathway for suspected treatment failure. Early in the assessment, clinicians should consider both persistence and reinfection; when feasible, apply genomic typing to distinguish them; and complement MIC testing with assays that capture killing kinetics, while recognising the methodological limitations of phenotypic tolerance testing. In our patient, prolonged ceftriaxone was chosen on the basis of his reporting no sexual contacts and previous success with this regimen in a previous case of tolerance-associated recurrent gonorrhoea. Sequencing, however, indicates that reinfection was the likely cause of his recurrent gonorrhoea. Routine extension of therapy should therefore be considered cautiously and always within a framework that addresses reinfection risk.

Our study has limitations. It is based on a single patient case, microbiological data from sexual partners were not available, and human pharmacokinetic measurements were not obtained. The *Galleria mellonella* model, while informative, does not fully recapitulate human gonococcal infection. In addition, the TD assay may be affected by experimental variables, including inoculum density, antibiotic carryover, and phase-variable phenotypic states, which could influence the interpretation of tolerance results. Whole-genome sequencing did not identify clear differences between tolerant and non-tolerant variants, but subtle genetic or regulatory differences cannot be fully excluded. The sequence types identified in this case were useful for distinguishing reinfection from persistence, but our single-patient dataset does not allow any inference as to whether a particular ST is more commonly associated with gonococcal disease burden or ceftriaxone tolerance. Isolate 25355 was selected for detailed phenotypic analysis because it was the most recent cultured isolate and, importantly, yielded both tolerant and non-tolerant subpopulations that enabled paired comparison within the same isolate background, rather than because its ST is known to be epidemiologically dominant or tolerance-associated. We did not perform transcriptomic profiling, as this was beyond the scope of the present study. Accordingly, whether differential gene expression contributed to the observed clinical course remains unresolved and warrants investigation in larger integrated genomic, phenotypic, and transcriptomic studies. Taken together, these findings across complementary *in vitro* and *in vivo* assays support the main interpretation of the study, while requiring confirmation in larger datasets.

In summary, tolerance can modulate killing kinetics and deserves attention in the evaluation of recurrent gonorrhoea, yet in this case the driver of recurrence was reinfection with genetically distinct strains. An evaluation approach that integrates prevention measures, retesting, genomic typing, and, where feasible, cautious phenotypic assessment of tolerance is likely to yield the most accurate diagnosis and the most effective care.

## Data Availability

The datasets presented in this study can be found in online repositories. The names of the repository/repositories and accession number(s) can be found below: https://www.ncbi.nlm.nih.gov/, PRJNA1334155.
