# Supplementary figures and images for: Reinfection and ceftriaxone tolerance in a clinical case of recurrent gonorrhoea: a case report supported by in vitro and in vivo models
Source: Front Cell Infect Microbiol. 2026 Apr 23;16:1720396. doi: 10.3389/fcimb.2026.1720396 (PMC13149363; doi:10.3389/fcimb.2026.1720396)

Supplementary Table 1: BUSCO output


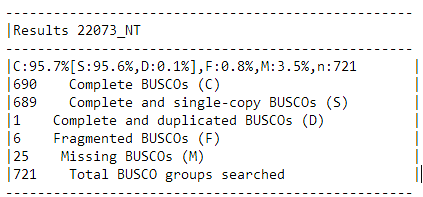


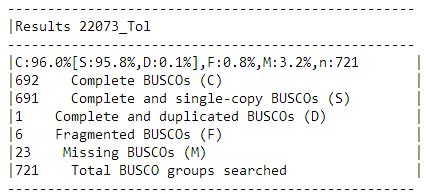


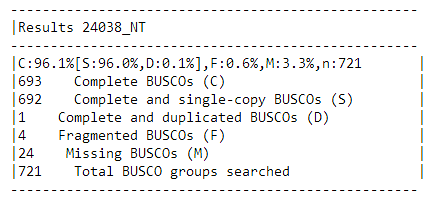


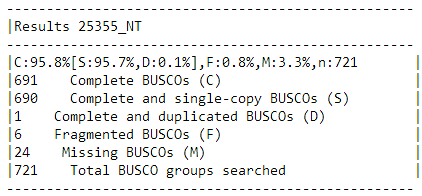


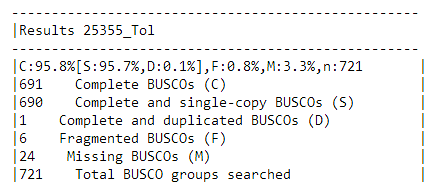


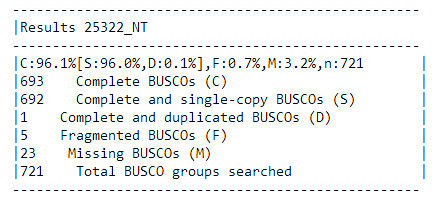

Supplement: Supplementary file 1 [file Table1.docx]
